# Right Ventricular Myxoma with a Papillary Muscular Origin

**DOI:** 10.3390/medicina60091390

**Published:** 2024-08-25

**Authors:** Kira Osipenko, Philipp Angleitner, Nikolaus Heinrich, Daniel Zimpfer, Martin Andreas

**Affiliations:** 1 Department of Cardiac Surgery, Medical University of Vienna, 1090 Vienna, Austria; 2 Division of Cardiothoracic and Vascular Anesthesia and Intensive Care Medicine, Department of Anesthesia, Critical Care and Pain Medicine, Medical University of Vienna, 1090 Vienna, Austria

**Keywords:** cardiac myxoma, right heart myxoma, right ventricular myxoma

## Abstract

Cardiac myxomas in the right ventricle are a very rare condition. In this case report, we describe an exceptionally uncommon case involving a right ventricular cardiac myxoma, originating from a papillary muscle, extending to both the tricuspid valve and the right atrium. The valve was able to be repaired via artificial chorda implantation.

## 1. Introduction

Cardiac myxomas are rare, benign neoplasms, observed in a small fraction of the general population [1] and accounting for approximately 50% of primary cardiac tumors [2]. Myxomas predominantly manifest in the left atrium. The right atrium is implicated in a notable, albeit lesser, proportion of these, ranging from 0.7% to 7.5%, while ventricles or heart valves are the most atypical locations in which these occur, with 0.7% to 3.6% and fewer than 1% of cases seen in these, respectively [3]. Here, we report a very rare case of right ventricular cardiac myxoma, with a papillary muscular origin, spreading to the tricuspid valve and right atrium.

## 2. Case Presentation

### 2.1. Clinical Presentation

A 52-year-old female patient presented with a history of intermittent chest discomfort and episodes of pre-syncope, palpitations, and vertigo over the past six months without dyspnea. The patient had no significant past medical history and no prior medication. The patient’s body mass index was 19.7 kg/m^2^.

### 2.2. Investigations

Despite the nonspecific nature of these symptoms, initial laboratory diagnostics, electrocardiogram, and cardiac MRI were performed to investigate the underlying cause of the patient’s symptoms.

The cardiac laboratory parameters were unremarkable, and the electrocardiogram demonstrated normal findings. 

**Figure 1:**
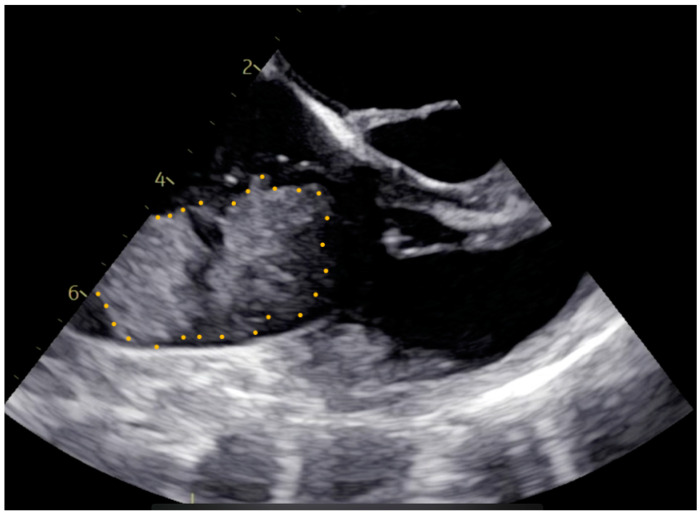
Pre-operative transesophageal echocardiogram image. The myxoma boundaries are delineated with a dotted yellow line.

**Figure 2:**
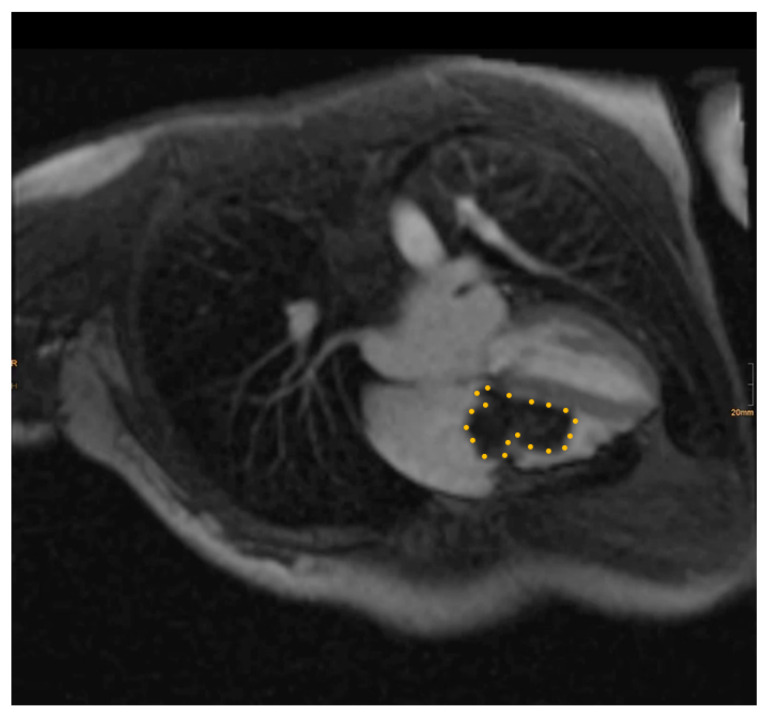
Pre-operative magnetic resonance image. The myxoma boundaries are delineated with a dotted yellow line.

Pre-operative cardiac magnetic resonance imaging (MRI) and transesophageal echocardiogram (TEE) revealed a myxoma measuring 46 mm × 37 mm × 30 mm in the right atrium. The base of the myxoma, located around the basal posterior wall of the right atrium, appeared to extend to the tricuspid valve and right ventricle (Figure 1 and Figure 2). Despite the localization and considerable size of the myxoma, there was no significant tricuspid valve insufficiency, and the function of both the left and right ventricles remained within normal parameters. The patient was evaluated for open-heart surgery involving the removal of a myxoma, with consideration for tricuspid valve replacement.

### 2.3. Management

**Figure 3:**
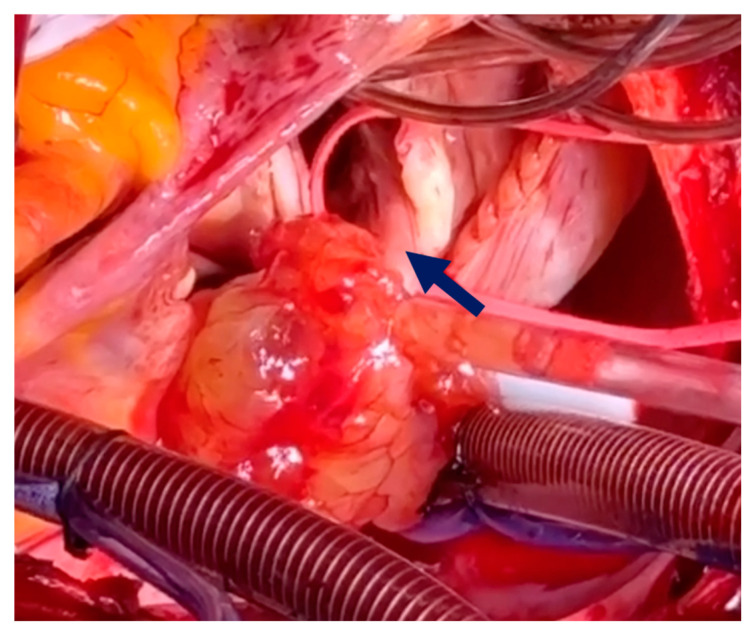
Cardiac myxoma extending toward the posterior leaflet chord. The blue arrow indicates the posterior leaflet chord.

The intervention was conducted with the patient under general anesthesia, with intubation and hemodynamic monitoring. The patient was fully heparinized, and cannulation was performed via arterial access through the ascending aorta and bicaval venous access. After the initiation of cardiopulmonary bypass, the right atrium was opened. The large myxoma originated from the apex of the papillary muscle, extending toward the posterior and septal leaflets, with chorda tendineae encroaching upon the posterior leaflet (Figure 3).

**Figure 4:**
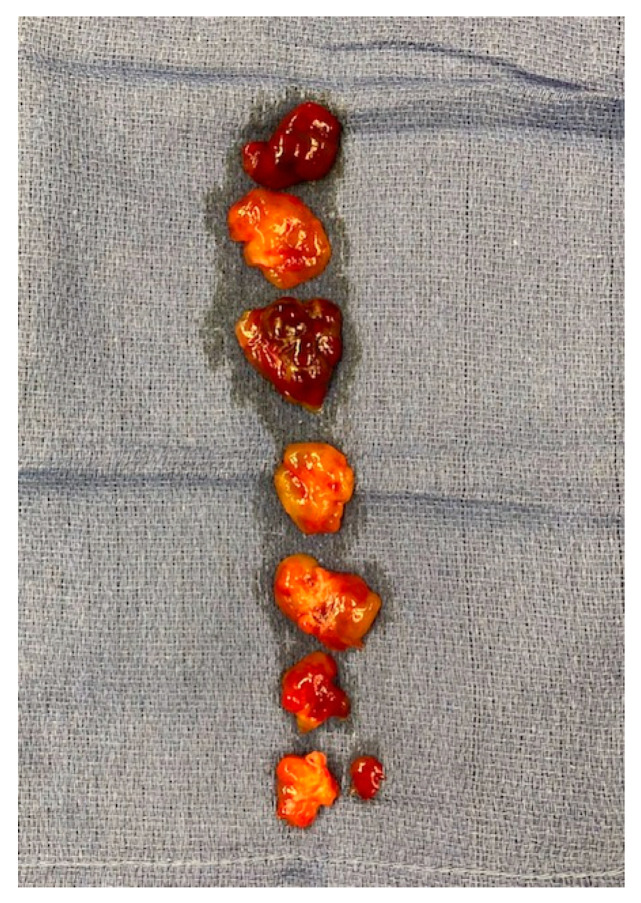
Fragments of myxoma after complete excision.

The resection, therefore, could only be accomplished in fragments (Figure 4). The tissue obtained during the operation was sent for histopathological examination, which confirmed the diagnosis of myxoma. 

**Figure 5:**
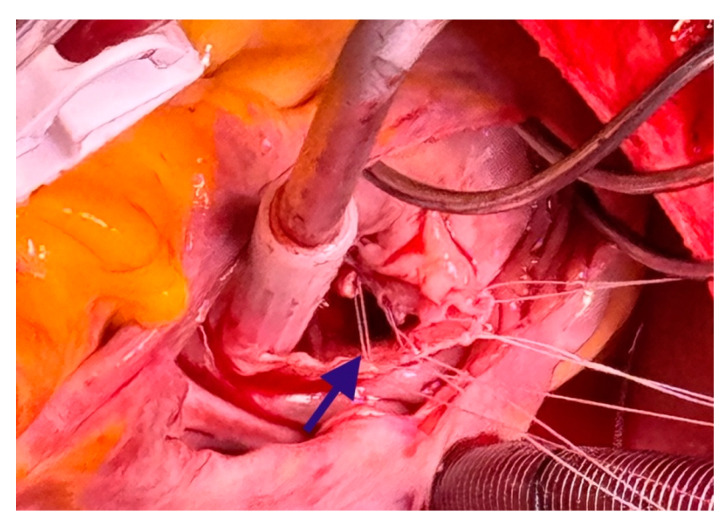
Placement of 18 mm Seramon loops (the blue arrow) onto the posterior papillary muscle.

**Figure 6:**
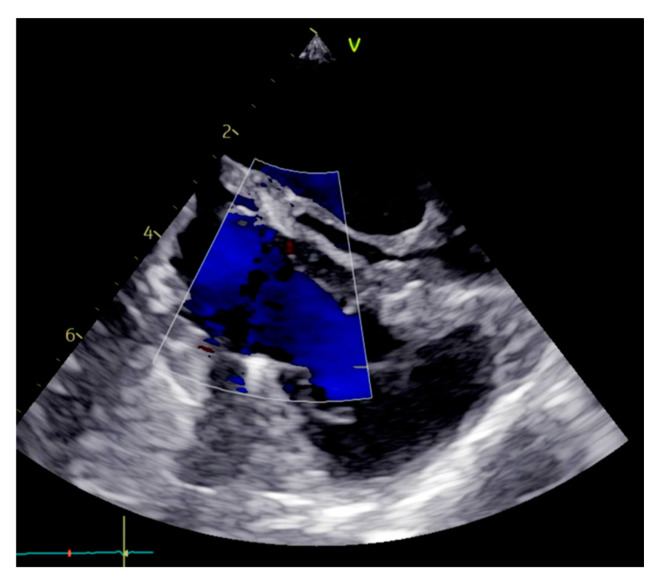
Post-operative transesophageal echocardiogram.

To achieve complete myxoma removal, the posterior leaflet chord was severed. Following the removal of the myxoma, the tip of the papillary muscle was resected. The tricuspid valve remained intact but exhibited insufficiency. The practitioners then decided to proceed with annuloplasty using a 30 mm Contour 3D annuloplasty ring (Medtronic, MN, USA). Subsequently, placement of 18 mm artificial chords (Seramon loops, Serag Wiessner, Naila, Germany) onto the posterior papillary muscle was carried out. The artificial chord was placed on the remnant non-fibrous part of the papillary muscle and two full-thickness bites were taken with pledgeted sutures. These four loops were then secured on the posterior and septal leaflets (Figure 5). The intraoperative echocardiography demonstrated valvular competence (Figure 6). The total duration of the operation was 198 min, with 70 min aortic of cross-clamping. During the procedure, the patient remained hemodynamically stable and was supported with 0.003 gamma noradrenaline. The patient was successfully extubated on the following day.

## 3. Post-Operative Course

As the patient remained pacemaker-dependent with a third-degree AV block on the 6th post-operative day, the decision was made to proceed with DDD-R pacemaker implantation with a quadripolar electrode on the left ventricle to spare the repaired tricuspid valve (Biotronik Amvia Sky HF-T QP, Berlin, Germany) [4].

To preserve right ventricular function, catecholamine support was tapered off until the fifth post-operative day. The patient was then discharged home with uneventful wound conditions on the ninth post-operative day. Pre-discharge echocardiography confirmed normal functioning of the tricuspid valve. The patient received enoxaparin sodium 4000 IU/0.4 mL for 4–6 weeks.

Six months post-operation, the patient is in a good general condition. Clinically, she exhibits no symptoms and has shown a favorable recovery trajectory. Follow-up assessments indicate stable cardiac function, with no signs of recurrence or complications related to the myxoma resection. Right ventricular dysfunction, which can be a serious complication, especially after right heart and isolated tricuspid interventions, was not observed [5]. Additionally, during the 3-month post-operative follow-up, there were no signs of AV blockages. Therefore, in the absence of AV block, pacemaker explantation might be considered in due course. The absence of symptoms such as dyspnea, chest pain, or palpitations suggests a successful surgical outcome and effective ongoing management.

## 4. Discussion

The primary objective of this case report was to elucidate an exceptionally rare case featuring an extensive cardiac myxoma and techniques to safe the native valve tissue. In our case, the myxoma was not localized in either the right or left atrium/ventricle; instead, it was in the right ventricle with an elongated attachment to the tricuspid valves, papillary muscles, and right atrium.

Despite the absence of associations between the localization and pathological profiles of cardiac myxomas and their clinical presentations, the variability in clinical manifestations remains high with nonspecific symptoms [6]. As a comprehensive diagnostic approach, encompassing echocardiography, cardiac magnetic resonance imaging is pivotal in establishing a diagnosis.

The main objective is to take a surgical approach as primary cardiac tumors continue to be a source of challenges in clinical contexts [1]. Typically, the standard procedure involves the complete removal of the myxoma in a single instance to avoid any loss of its components. This unique manifestation of cardiac myxoma posed a surgical challenge due to its extension into the papillary muscles. Excising all components of the myxoma necessitated an incision into one papillary muscle, making the resection of the myxoma feasible only in a partial, sequential manner. Most importantly, especially in young patients, repair of the tricuspid valve should be targeted whenever possible. As all chords from the papillary muscle had to be resected to remove the tumor completely, artificial chord placement was the only option to preserve the native valve. Correct length assessment and additional annuloplasty are crucial to regaining normal valve function. Tricuspid valve repair with artificial chords is more challenging compared to that on the left side, as three instead of two leaflets have to coapt. If no sufficient results are achieved with the artificial chord technique, the clover technique might be a good alternative [7]. Furthermore, pacemaker lead placement through a repaired tricuspid valve should be avoided according to current recommendations, and alternatives are available [4].

## 5. Conclusions

Right-sided myxomas are relatively uncommon. This case of an extensive cardiac myxoma, with a papillary muscular origin, presents a unique clinical scenario, which required specific surgical techniques to allow valve repair. It highlights the crucial role of advanced imaging in diagnosis, the need for personalized surgical planning, and the potential for successful outcomes even in complex cases. Preserving the native valve tissue when possible and carefully using artificial chords are key strategies for achieving good surgical results. The patient’s excellent recovery and ongoing stability show that the surgical and post-operative strategies were effective, offering hope for similar cases in the future.

## Data Availability

Data are available in a repository and can be accessed via a DOI link.
